# Database-based Eco-Plant analysis for Mesozoic dispersed sporomorphs

**DOI:** 10.1016/j.mex.2021.101329

**Published:** 2021-04-05

**Authors:** Jianguang Zhang, Olaf Klaus Lenz, Pujun Wang, Youfeng Gao, Jens Hornung

**Affiliations:** a Technische Universität Darmstadt, Schnittspahnstraße 9, Darmstadt 64287, Germany; b Senckenberg Gesellschaft für Naturforschung, General Directorate, Senckenberganlage 25, 60325, Frankfurt/Main, Germany; c Key Laboratory for Evolution of Past Life and Environment in Northeast Asia (Jilin University), Ministry of Education, Changchun 130026, China; d College of Earth Sciences, Jilin University, Changchun 130061, PR China

**Keywords:** Botanical affinity, Ecogroup, Palaeoenvironment, Palaeoclimate, Palaeoecology, Sporopollen

## Abstract

Patterns of community assemblage for plants are associated with particular climatic elements such as water, heat, light, and air. The classification based on these plant assemblages is referred to here as the ecogroup of plants (Eco-Plant), whereas the method of analysing palaeoenvironmental and palaeoclimate variation by using Eco-Plant is called the Eco-Plant model. The online database *Sporopollen* was created to quickly assign eco-climatic traits to quantitative fossil sporomorph data to assess implications for past vegetation patterns and climatic changes. A user-friendly interface has been created, where users can upload their data to the database and in return get immediate results. This database can automatically link all Mesozoic and Cenozoic sporomorphs to their putative parent plants at phylum, order, or family level. It can also automatically link all Triassic and Jurassic sporomorphs to Eco-Plant groups to assess the effect of humidity (EPH) and the effect of temperature (EPT).

• The Eco-Plant model allows to reconstruction of relative Triassic and Jurassic humidity and temperature changes.
• A useful tool for palaeoenvironmental reconstruction.
• A useful tool for (high-resolution) palynological studies.

## Graphical abstract

**Figure:**
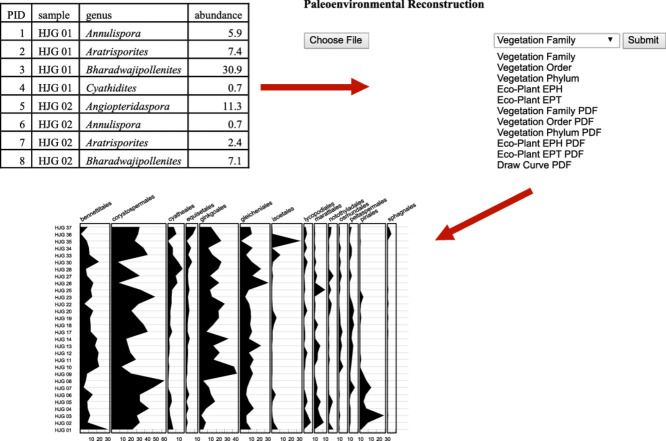

Specifications Table

**Table:** 

Subject Area:	Palynology
More specific subject area:	Mesozoic Palynology
Method name:	***Sporopollen***
Name and reference of original method:	No
Resource availability:	***Sporopollen*** http://www.sporopollen.com/sporemesozoicsegs.php?opencode=paper1

## Method details

### Background

The seminal work of Warming [1] and Schimper [2] recognized that patterns of community assemblage for plants were associated with particular climatic elements such as water, heat, light, and air. The classification based on these plant assemblages is referred to here as the ecogroup of plants (Eco-Plant), whereas the method of analyzing palaeoenvironmental and palaeoclimate variation by using Eco-Plant is called the Eco-Plant model [3]. The term is used to distinguish the model from the Sporomorph Ecogroup Model (SEG model) created by Abbink et al. [4]. The Eco-Plant model has been widely used by palaeobotanists for extant (e.g., [5], [6], [7], [8]), Cenozoic (e.g., [9], [10], [11]), Mesozoic (e.g., [12–13]), and Paleozoic (e.g., [14–15]) palaeoenvironmental reconstructions. It is also applied by palynologists for palaeoenvironmental reconstructions using dispersed sporomorphs from the Cenozoic (e.g., [16], [17], [18], [19]) and Mesozoic (e.g., [3,[20], [21], [22], [23], [24], [25], [26], [27]).

Previously, for most of the Mesozoic dispersed sporomorphs, the application of the Eco-Plant model was limited, because either their assignment to a specific Eco-Plant was uncertain or the botanical affinities to natural plant taxa were unclear. It was thus important to identify the botanical affinities of Mesozoic dispersed sporomorphs, to provide reliable assignment to an Eco-Plant [3].

The online database ***Sporopollen*** (http://www.sporopollen.com) was created to link the dispersed sporomorphs (spores and pollen) to past vegetation patterns and climatic changes. The goal of the database is to analyze sporomorph data, especially of Mesozoic sporomorphs, for identification of parent plants, stratigraphic analysis, and to aid palaeoenvironmental reconstruction. This paper only focuses on the algorithm for palaeoenvironmental reconstruction, which automatically assigns eco-climatic traits to fossil sporomorph taxa that are uploaded by users. This can be used without the need to log in at http://www.sporopollen.com/sporemesozoicsegs.php?opencode=paper1. The sporomorph data published by Zhang et al. [3] are used as examples to show how the database works. The implementation of the Eco-Plant model connects Mesozoic sporomorphs to their most likely plant taxa and their associated temperature and humidity groups (details below). The output varies depending on the options selected but serves as an aid for the researcher who must still interpret the relative abundances of taxa from different Eco-Plant groups in the samples.

### Database concept

**Fig. 1:**
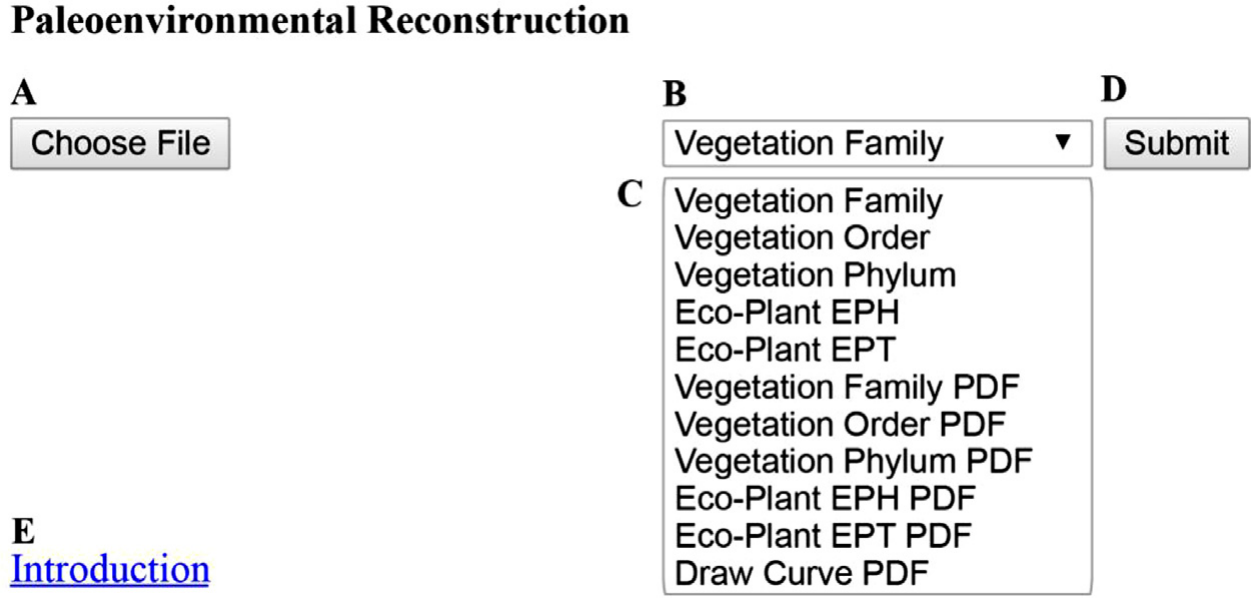
User-friendly interface for palaeoenvironmental reconstruction is provided by ***Sporopollen***. **A.** Choose File button, by click users can upload their data to the database. Currently, only CSV file is accepted; **B.** Drop-down list, by click users can select different modes of palaeoenvironmental reconstruction; **C.** Different modes of palaeoenvironmental reconstruction; **D.** Submit button, by click users can quickly get the result, when a CSV file is uploaded and the palaeoenvironmental reconstruction mode is selected; **E.** Introduction links, by click users can get the detailed manual and example files. It can be visited at: http://www.sporopollen.com/sporemesozoicsegs.php?opencode=paper1. Manual and example files can be visited at: http://www.sporopollen.com/sporeexample.php?opterate=Paleoenvironmental%20Reconstruction.

The database system uses MySQL [28] for the database server and Hypertext Preprocessor (PHP) [29] for the dynamic website. A user-friendly interface (Fig. 1) has been created, which automatically assigns eco-climatic traits to the uploaded fossil sporomorph data (Appendix 1 for PHP code). In the database, datasets *Taxonomy* (Table 1) and *Ecogroup* (Table 2) are created. In dataset *Taxonomy* (Table 1), six fields *Kingdom, Phylum, Class, Order, Family*, and *Genus* are used to store the taxonomic ranks for both plant and sporomorph genera. In dataset *Ecogroup* (Table 2), three fields *Eph, Ept*, and *Genus* are used to store the Eco-Plant information. As different authors may have slightly different views on the concept of Eco-Plant groups (e.g., [20–21]), in this database, the concept that assesses the effects of humidity (EPH) and temperature (EPT) follows Zhang et al. [3] and Zhang [30]. The Eco-Plant groups separate hydrophytes, hygrophytes, mesophytes, xerophytes, and euryphytes based on their adaptation to different humidities (EPH). Additionally, megathermic, mesothermic, microthermic, and eurythermic plants are distinguished based on their adaptation to different temperatures (EPT).

**Table 1:** Part of the dataset Taxonomy storing data of the taxonomic ranks.

Kingdom	Phylum	Class	Order	Family	Genus
Plantae	Gymnospermae		Bennettitales	Williamsoniaceae	*Bharadwajipollenites*
Plantae	Gymnospermae		Bennettitales	Cycadeoidaceae	*Huabeisporites*
Plantae	Gymnospermae		Corystospermales	Umkomasiaceae	*Pteruchipollenites*
Plantae	Pteridophytes	Polypodiopsida	Cyatheales	Cyatheaceae	*Cyathidites*
Plantae	Pteridophytes	Polypodiopsida	Cyatheales	Cibotiaceae	*Duplexisporites*
Plantae	Pteridophytes	Equisetopsida	Equisetales	Equisetaceae	*Calamospora*
Plantae	Gymnospermae		Ginkgoales	Ginkgoaceae	*Ginkgocycadophytus*
Plantae	Pteridophytes	Polypodiopsida	Gleicheniales	Dipteridaceae	*Dictyophyllidites*
Plantae	Pteridophytes	Lycopodiopsida	Isoetales	Pleuromeiaceae	*Aratrisporites*
Plantae	Pteridophytes	Lycopodiopsida	Lycopodiales	Lycopodiaceae	*Hamulatisporis*
Plantae	Pteridophytes	Lycopodiopsida	Lycopodiales	Lycopodiaceae	*Foveolatitriletes*
Plantae	Pteridophytes	Marattiopsida	Marattiales	Marattiaceae	*Angiopteridaspora*
Plantae	Bryophytes	Anthocerotopsida	Notothyladales	Notothyladaceae	*Annulispora*
Plantae	Pteridophytes	Polypodiopsida	Osmundales	Osmundaceae	*Osmundacidites*
Plantae	Gymnospermae		Peltaspermales	Peltaspermaceae	*Protohaploxypinus*
Plantae	Gymnospermae		Pinales	Pinaceae	*Pinuspollenites*
Plantae	Gymnospermae		Pinales	Cheirolepidiaceae	*Discisporites*

**Table 2:** Part of the dataset Ecogroup storing data of Eco-Plant groups that assess the effect of humidity (EPH) and the effect of temperature (EPT).

Genus	EPH	EPT
*Angiopteridaspora*	hygrophytes	megathermic
*Annulispora*	hygrophytes	eurythermic
*Aratrisporites*	hydrophytes	eurythermic
*Calamospora*	hygrophytes	eurythermic
*Cyathidites*	hygrophytes	megathermic
*Dictyophyllidites*	mesophytes	megathermic
*Discisporites*	xerophytes	megathermic
*Duplexisporites*	hygrophytes	megathermic
*Foveolatitriletes*	hygrophytes	eurythermic
*Ginkgocycadophytus*	mesophytes	mesothermic
*Hamulatisporis*	hygrophytes	eurythermic
*Huabeisporites*	hygrophytes	megathermic
*Osmundacidites*	hygrophytes	eurythermic
*Pinuspollenites*	mesophytes	microthermic
*Protohaploxypinus*	xerophytes	megathermic
*Pteruchipollenites*	mesophytes	megathermic

In the database, the taxonomic ranks of extant plants mainly follow Christenhusz et al. [31], Goffinet and Buck [32], and Smith et al. [33], the taxonomic ranks of fossil plants mainly Taylor et al. [34] and the taxonomic ranks of Mesozoic fossil pollen of angiosperms mainly Song et al. [35] and Muller et al. [36]. By comparing the unique outline and structure/sculpture of the sporomorph wall with that of modern plants and *in situ* fossil plants (plants with spores and pollen grains *in situ* within a sporangium), 859 dispersed Mesozoic sporomorph genera of Bryophytes, Pteridophytes, and Gymnosperms are reviewed [30]. The illustrations, descriptions, and definitions of dispersed sporomorphs are mainly after Jiang et al. [37], Huang [38], Liu [39], Shang [40], Song et al. [41], Song et al. [42], Shu and Norris [43], the 6 vol book series of “*Synopsis der Gattungen der Sporae dispersae*” [44], [45], [46], [47], [48], [49], and the 26 vol book series of “*Catalog of fossil spores and pollen*” [50], [51], [52], [53], [54], [55], [56], [57], [58], [59], [60], [61], [62], [63], [64], [65], [66], [67], [68], [69], [70], [71], [72], [73], [74]. Illustrations and descriptions of extant sporomorphs are mainly after Zhang et al. [75], Wang and Dai [76], Li et al. [77], Tryon and Lugardon [78], Hesse et al. [79], Boros and Járai-Komlódi [80], and Kramer and Green [81]. To date, among the 859 dispersed Mesozoic sporomorph genera 484 of them can be linked to their closest parent plants and Eco-Plant groups at family or order level. Because of the lack of detailed ultrastructure descriptions or unclear separation to other genera 375 of 859 dispersed Mesozoic sporomorph genera cannot be linked at the moment to any parent plants and Eco-Plant groups [30]. For each genus that can be linked to its closest parent plant, the most likely plant family is stored in the dataset *Taxonomy*. For 484 of 859 dispersed Mesozoic sporomorph genera, their EPH and EPT are reviewed [30] and stored in the dataset *Ecogroup* (Table 2).

**Fig. 2:**
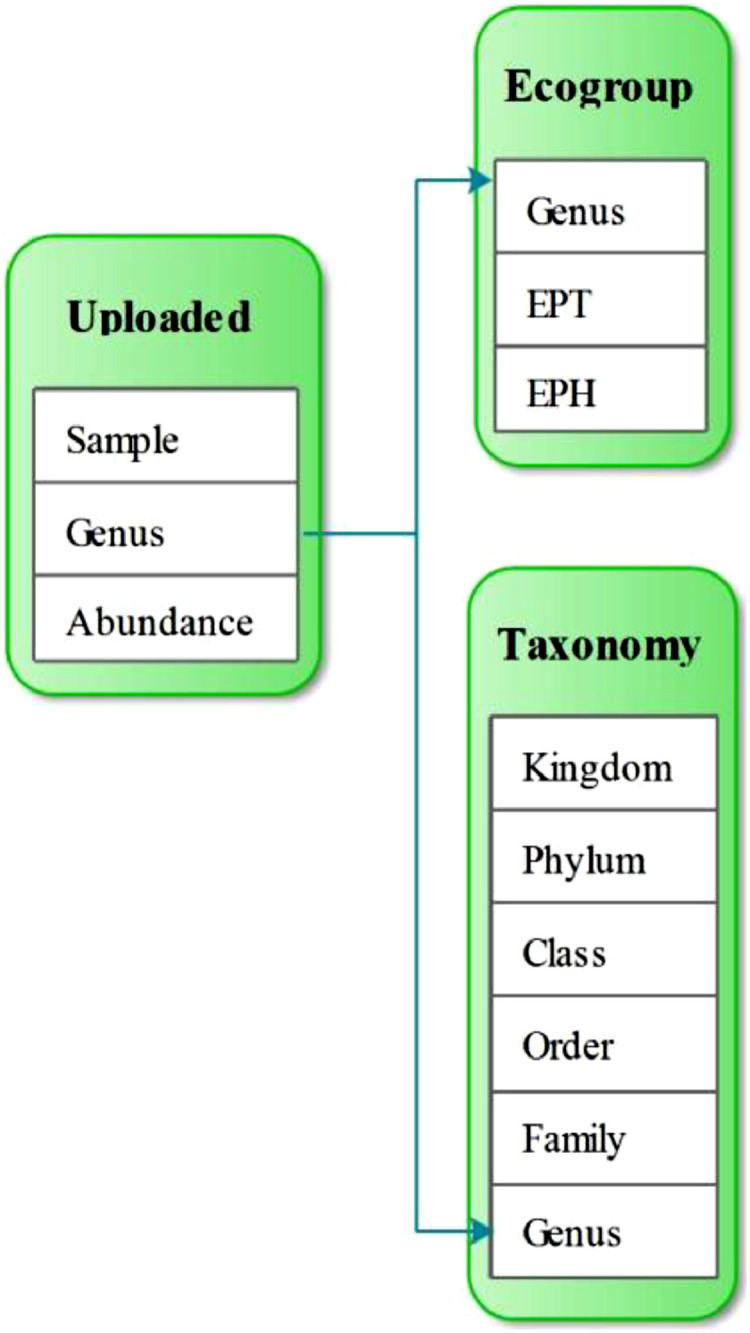
Relationship of the datasets *Taxonomy* (Table 1) and *Ecogroup* (Table 2) in the database and the user-uploaded dataset (Table 3). All the datasets have the same field of Genus, by the same genera stored in the fields different datasets can be integrated as a single dataset.

The dataset that will be uploaded by users must include three fields, which are *Sample, Genus*, and *Abundance* (Table 3). The data in field *Abundance* can be either the number of counted grains (raw data) or percentage values of the sporomorphs sorted by sample. Due to the same genus in both the uploaded dataset and the dataset *Taxonomy* (Table 1), the abundances for Phylum, Order, and Family in each of the samples are calculated. Furthermore, based on the same genus in both the uploaded dataset and the dataset *Ecogroup* (Table 2), the abundances of EPH and EPT are provided (Fig. 2 for dataset relationship).

**Table 3:** Part of the example uploaded file which can be used for all the analysis modes except Draw Curve PDF.

Depth	Sample	Genus	Abundance
1	HJG 01	*Annulispora*	5.9
1	HJG 01	*Aratrisporites*	7.4
1	HJG 01	*Bharadwajipollenites*	30.9
1	HJG 01	*Cyathidites*	0.7
1	HJG 01	*Dictyophyllidites*	13.2
1	HJG 01	*Discisporites*	1.5
1	HJG 01	*Duplexisporites*	5.9
1	HJG 01	*Ginkgocycadophytus*	4.4
1	HJG 01	*Huabeisporites*	2.9
1	HJG 01	*Osmundacidites*	0.7
1	HJG 01	*Pteruchipollenites*	25
1	HJG 01	*Quadraeculina*	1.5
2	HJG 02	*Angiopteridaspora*	11.3
2	HJG 02	*Annulispora*	0.7
2	HJG 02	*Aratrisporites*	2.4
2	HJG 02	*Bharadwajipollenites*	7.1
2	HJG 02	*Calamospora*	2
2	HJG 02	*Cyathidites*	4
2	HJG 02	*Dictyophyllidites*	7.7
2	HJG 02	*Discisporites*	9.1
2	HJG 02	*Duplexisporites*	0.7
2	HJG 02	*Foveolatitriletes*	5.5
2	HJG 02	*Ginkgocycadophytus*	7.5
2	HJG 02	*Hamulatisporis*	2.9
2	HJG 02	*Huabeisporites*	1.8
2	HJG 02	*Pinuspollenites*	0.7
2	HJG 02	*Protohaploxypinus*	1.3
2	HJG 02	*Pteruchipollenites*	34
2	HJG 02	*Quadraeculina*	1.5

**Note:** This file must be saved as a CSV file. The field names can be lower case, upper case, and mixed case. The data in the abundance field can be both percentage value and the counted number of the respective grains.

The full file can be downloaded at: http://www.sporopollen.com/sporeexample.php?opterate=Paleoenvironmental%20Reconstruction.

**Fig. 3:**
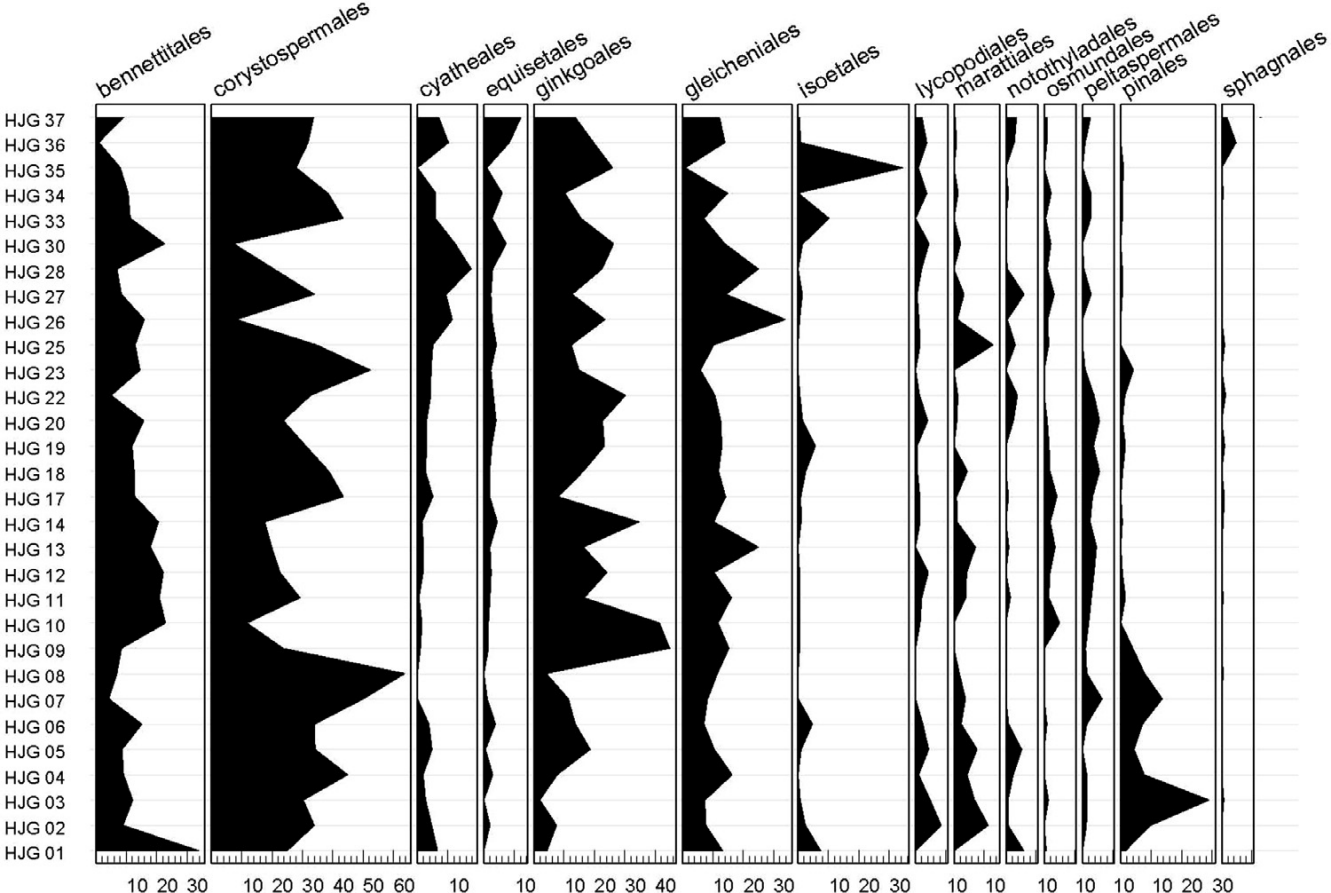
The result can be output in the form of a diagram showing the percentages of plant orders. It is the result of user-uploaded dataset (Table 3) connected with the dataset Taxonomy (Table 1) with the analysis mode of Vegetation Order PDF. All of the abundances are in percentages.

The uploaded dataset, strictly following the format of Table 3, must be saved as a CSV file which is a common way to transfer datasets between different databases. Then the interface at http://www.sporopollen.com/sporemesozoicsegs.php?opencode=paper1 is needed (Fig. 1). Interface manuals and example files can be visited by clicking *Introduction* (Fig. 1-E), which is linked to http://www.sporopollen.com/sporeexample.php?opterate=Paleoenvironmental%20Reconstruction. By clicking the *Choose File* button (Fig. 1-A), a window will pop up for users to select the uploaded dataset stored in the local computer. The name of the button will be different if the user has a non-English system. For example, in the Chinese system, the button will be shown with Chinese characters. When the uploaded dataset is chosen, one of the analysis modes needs to be selected by clicking the *select box* (Fig. 1-B). There are different modes available (Fig. 1-C). The modes of *Vegetation Family, Vegetation Order*, and *Vegetation Phylum* will link the sporomorph genera in the uploaded dataset to their parent plant separately at family, order, and phylum level. The modes of *Eco-Plant EPH* and *Eco-Plant EPT* will link the sporomorph genera in the uploaded dataset separately to EPH and EPT. The results of all five modes will be shown in the form of a dataset yielding the abundances, (Table 4) which are calculated as percentages sorted by samples. If the results mentioned above shall be presented are needed as a graphical diagram (Fig. 3), the modes of *Vegetation Family PDF, Vegetation Order PDF, Vegetation Phylum PDF, Eco-Plant EPH PDF*, or *Eco-Plant EPT PDF* should be selected. Users may want to combine the results of different modes into one diagram or delete some fields from the diagram. Therefore, the mode of *Draw Curve PDF* (Fig. 1-C) should be selected to use this function. In this case, the uploaded dataset must follow the format presented in Table 5 and also be saved as a CSV file. The first column of the uploaded dataset must be named *Sample* and includes the sample names or numbers. The other columns must be used to store the abundances of the palynomorphs in percentages (Table 5).

**Table 4:** The result for Vegetation Order using the data in Table 3.

Sample	HJG 01	HJG 02
Bennettitales	33.8	8.9
Corystospermales	25	33.9
Cyatheales	6.6	4.7
Equisetales	0	2
Ginkgoales	4.4	7.5
Gleicheniales	13.2	7.7
Isoetales	7.4	2.4
Lycopodiales	0	8.4
Marattiales	0	11.3
Notothyladales	5.9	0.7
Osmundales	0.7	0
Peltaspermales	0	1.3
Pinales	1.5	9.8
Uncertain	1.5	1.5

**Note:** This result is based on the combination of the datasets Taxonomy (Table 1) and the uploaded dataset (Table 3) by the same genera in the fields of Genus in both datasets. The result abundances are percentages sorted by samples. Genera with uncertain affinities are marked as Uncertain.

**Table 5:** Part of the example file accepted by ***Sporopollen*** which can be used only for Draw Curve PDF.

Sample	Bennettitales	Cheirolepidiaceae	Corystospermales	Cyatheales
HJG 01	33.8	1.5	25	6.6
HJG 02	8.9	9.1	34	4.7
HJG 03	12.1	29.1	30.4	2.7
HJG 04	9.1	7.2	45	1.9
HJG 05	8.6	2.9	34.3	4.8
HJG 06	15.1	5.7	34	3.8
HJG 07	4.2	9.8	50	0
HJG 08	6.9	0.9	63.8	0

**Note:** This file must be saved as a CSV file. The field names can be lower case, upper case, and mixed case. The first field of the uploaded dataset must be Sample which stores the sample names. The other fields must be used to store the abundances in percentages.

The full file can be downloaded at: http://www.sporopollen.com/sporeexample.php?opterate=Paleoenvironmental%20Reconstruction.

**Fig. 4:**
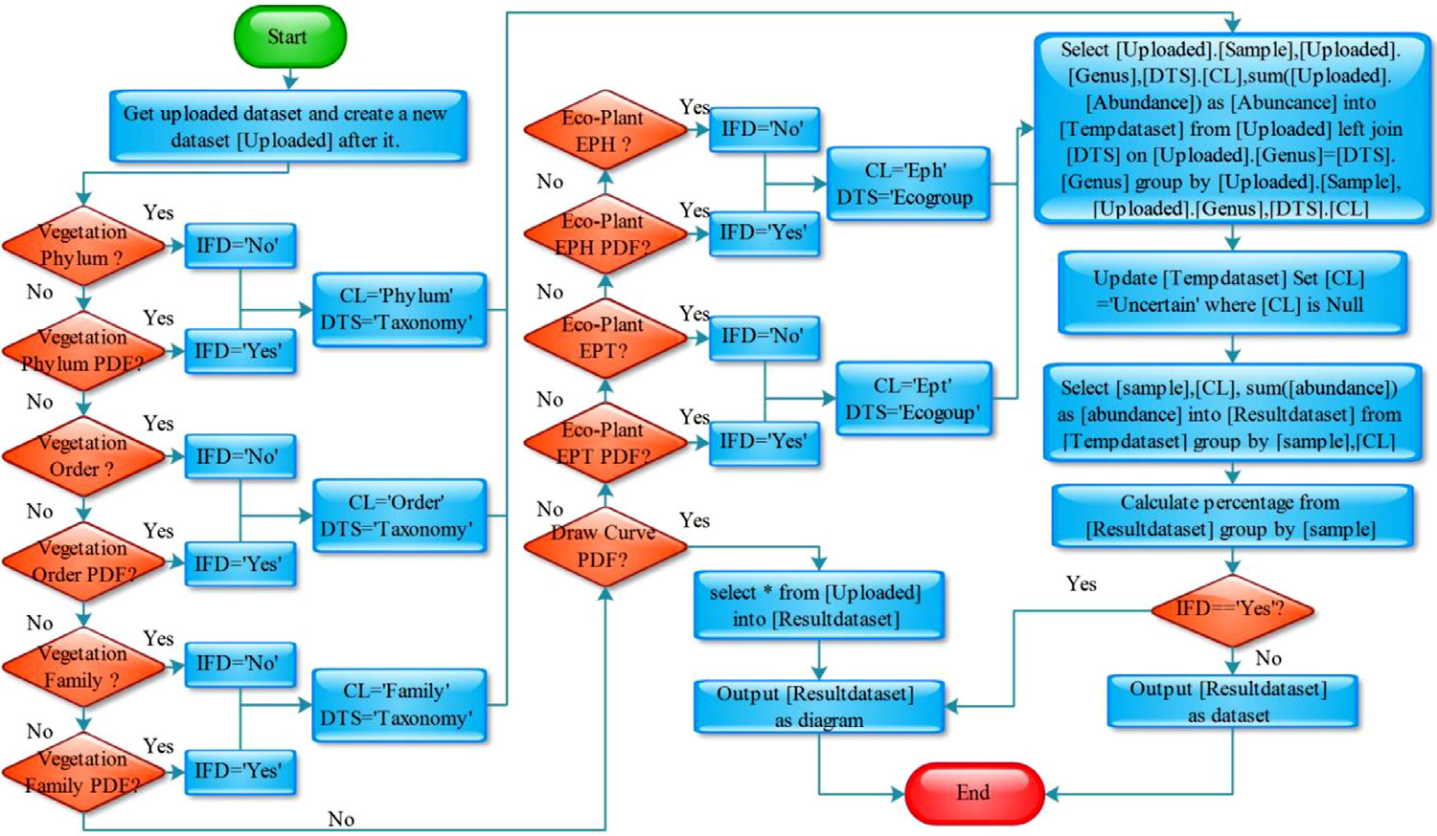
Flowchart for palaeoenvironmental reconstruction. Dataset names and attribute names are in square brackets, e.g., [Sample]. Dataset and field names are in title, e.g., Uploaded and Phylum. Variables are in upper case e.g., CL, DTS, IFD. [Uploaded] is the dataset storing the analysed data uploaded by users; [Tempdataset] is temporary dataset; [Resultdataset] is the result dataset. In this paper [Uploaded] is Table 3 or 5, [Taxonomy] is Table 1, and [Ecogroup] is Table 2.

When the uploaded dataset and analysis mode are chosen, by clicking the *Submit* button (Fig. 1-D) the user immediately gets the result. MySQL combined with PHP codes are used to automatically finish the process (Fig. 4 for flowchart; Appendix 2 for PHP and MySQL codes). Based on the codes, the first step of processing the data is to receive the user-uploaded dataset and store it as a new dataset *Uploaded*. Afterwards, based on the chosen process modes, abundances are calculated (Fig. 4).

If the mode *Vegetation Phylum, Vegetation Phylum PDF, Vegetation Order, Vegetation Order PDF, Vegetation Family*, or *Vegetation Family PDF* is selected by the user, the user-uploaded dataset will automatically be linked to the dataset *Taxonomy* producing a combined dataset *Tempdataset*. If the mode is *Vegetation Phylum* or *Vegetation Phylum PDF*, abundance values are calculated by grouping the data by the fields *Sample* and *Phylum*. In contrast, if the selected mode is *Vegetation Order* or *Vegetation Order PDF*, abundances are calculated by grouping the data by the fields *Sample* and *Order*. Finally, if the chosen mode is *Vegetation Family* or *Vegetation Family PDF*, abundances are calculated by grouping the data by the fields *Sample* and *Family*. The genera without botanical affinity will be marked as *Uncertain* in all the six modes. For the modes *Vegetation Phylum, Vegetation Order*, and *Vegetation Family*, the output is a dataset of abundance data, for the modes *Vegetation Phylum PDF, Vegetation Order PDF*, and *Vegetation Family PDF*, the user will get a diagram of abundance data comparable to a pollen diagram.

If the mode *Eco-Plant EPH, Eco-Plant EPH PDF, Eco-Plant EPT*, or *Eco-Plant EPT PDF* is selected by the user, the uploaded dataset will automatically be linked to the dataset *Ecogroup* producing a combined dataset *Tempdataset*. If the mode is *Eco-Plant EPH* or *Eco-Plant EPH PDF*, abundances are calculated by grouping the data by the fields *Sample* and *Eph*. In contrast, if the chosen mode is *Eco-Plant EPT* or *Eco-Plant EPT PDF*, abundances are calculated by grouping the data by the fields *Sample* and *Ept*. Palynomorph genera without botanic affinity are marked as *Uncertain* in all the modes. For the modes of *Eco-Plant EPH* and *Eco-Plant EPT*, the output is a dataset of abundance data, whereas for the modes of *Eco-Plant EPH PDF* and *Eco-Plant EPT PDF* the user will get a diagram of abundance data comparable to a pollen diagram.

If the user selects *Draw Curve*, the user-uploaded dataset will be directly plotted as a diagram without linking to any database dataset.

## Discussion

The Sporomorph Ecogroup Model (SEG model) of Abbink et al. [4] is commonly used for assigning eco-climatic traits to Mesozoic fossil sporomorph taxa of Europe and some parts of China (e.g., [4,[82], [83], [84], [85], [86]). It represents a simplified Eco-Plant model. According to hydrologic and temperature conditions in the Eco-Plant model, plants are classified into different EPH and EPT groups due to their climatic preferences. In contrast, in the SEG model, plants are classified as belonging to a wetter, drier, warmer, or cooler group. Besides, in the SEG model, due to uncertain botanical affinities of some palynomorphs, several plants indicating a different climate and environment are categorized in the same group. For example, in the Eco-Plant model, Ginkgoales are classified as mesophytes and mesothermic plants, but Bennettitales as hygrophytes and megathermic plants [3]. In contrast, in the SEG model, Ginkgoales, Cycadales, and Bennettitales are all included in the same group of the “Lowland SEG” and indicate a “drier” and “warmer” climate, since the pollen of Ginkgoales, Cycadales, and Bennettitales can usually only be distinguished under scanning electron microscopy (SEM) or transmission electron microscope (TEM) [4]. Therefore, the Eco-Plant model is chosen in our database since it allows for more detailed and precise statements on palaeoclimate than the SEG model.

Zhang et al. [3] only linked 19 sporomorph genera to 15 families or orders of Mesozoic plants and Eco-Plant. However, in this database, many more genera are included in dataset *Taxonomy* (Table 1). Fossil sporomorph taxa from the Mesozoic to extant can be linked to their parent plants at family, order, or phylum level by the database.

The Eco-Plant model is applied by palynologists for palaeoenvironmental reconstructions using dispersed sporomorphs from the Cenozoic (e.g., [16–19]) and Mesozoic (e.g., [3,20–27]). However, for most of the Mesozoic dispersed sporomorphs, the application of Eco-Plant is limited, because either their assignment to a specific Eco-Plant remains uncertain or the botanical affinities to plant taxa are unclear [3]. For the dispersed Mesozoic sporomorph genera of Bryophytes, Pteridophytes, and Gymnosperms [30], which are included in the database, their EPH and EPT are stored in dataset *Ecogroup* (Table 2). The dispersed sporomorphs of angiosperms are currently not linked to Eco-Plant. Nevertheless, the first reliable angiosperm is discovered at the top of the Jurassic [87–88]. Therefore, this database is capable of automatically linking all sporomorph genera from the Triassic and the Jurassic to Eco-Plant to reconstruct relative climate changes. However, the terms used by different authors for Eco-Plant groups are not always the same, making the comparison of results between different authors difficult, e.g., the *Thermophytic form* used by Wang et al. [20] is more or less the same as the *Megathermic element* used by Suc and Fauquette [19]. The term *Halophytes* is equivocal [89] and it is difficult to distinguish this Eco-Plant group from *Xerophytes.* Therefore, most authors (e.g., [20,22]) use the term *Xerophytes* to represent both *Halophytes* and *Xerophytes* in the Mesozoic. In this database, the concept of Eco-Plant groups that assess the effect of humidity (EPH) and the effect of temperature (EPT) follows Zhang et al. [3] and Zhang [30], which makes it easier to compare the results between different users using the same concepts. Users can also review published sporomorph data by extracting them from published studies and by uploading these data to the database.

The database provides a tool for linking dispersed sporomorph genera to their parent plants and analysing them for palaeoecological and palaeoclimatic implications. Usually, to get for a user-uploaded dataset the result of the analyses, only several seconds are needed. High-resolution palynomorph studies usually need to process huge amount of sporomorph data. This database can speed up the process and save time for users. The obtained result in the form of a dataset with abundance values (e.g., Table 4) can be used for, e.g., further statistical analysis by other software, e.g., PAST [90], or for producing diagrams by other programs e.g., PanPlot 11.04 [91]. The graphical output in form of a diagram (e.g., Fig. 3) gives the user a quick and vivid overview of the results.

The algorithm that assigns eco-climatic traits to the uploaded fossil sporomorph data (Fig. 4) and the database datasets (Tables 1 and 2) are independent, which allows an easy database update. In conclusion, if there are new published studies on botanical affinities of Paleozoic and Mesozoic sporomorph taxa, the related information only needs to be included in the dataset *Taxonomy* (Table 1). If there are, e.g., new studies on the EPH and EPT of dispersed sporomorphs for angiosperms, only the dataset *Ecogroup* (Table 2) must be updated. Furthermore, when there are new algorithms needed to process the sporomorph taxa, only the new PHP and MySQL codes must be compiled and linked with the user interface (Fig. 1). In future, any information on the modification of the database will appear below the user interface (Fig. 1). As it is an online database, any new changes in this database will have an immediate benefit for all users.

To produce reliable results, data on botanical affinities of sporomorphs and environmental information for the parent plants stored in the datasets *Taxonomy* (Table 1) and *Ecogroup* (Table 2) are based on published studies. However, systematic analysis is needed when there are uncertainties, e.g., on botanical affinities, due to different affinities published in different studies. As an example, *Quadraeculina* Maljavkina, 1949 ex Potonie 1960 is related to the Podocarpaceae [4] or Caytoniaceae [92], etc. However, no reliable *in situ* pollen (pollen grains *in situ* within a sporangium) has been published. In this case, the affinity of *Quadraeculina* is marked as *Uncertain* in the dataset *Taxonomy. Annulispora* De Jersey, 1959 McKellar 1947 was reported to be related to Sphagnaceae [93], but based on scanning electron microscope (SEM) studies, the spore is more comparable with the extant spores of Notothyladaceae [3]. In this case, the affinity of *Annulispora* is marked as Notothyladaceae in the dataset *Taxonomy*. Nevertheless, all studies on botanical affinities for the collected sporomorph genera in dataset *Taxonomy* can be viewed by adding a single genus name in the text box combined with clicking the *Search* button at http://www.sporopollen.com/sporeidentify.php?operate=taxonomy&taxonomy=Annulispora or by selecting the genus name at http://www.sporopollen.com/sporefamilygenus.php?language=english. By comparing the unique outline and structure/sculpture of the sporomorph wall with that of pollen/spores of modern plants and *in situ* fossil plants, the botanical affinities, EPH, and EPT of the 859 dispersed Mesozoic sporomorph genera of Bryophytes, Pteridophytes, and Gymnosperms are reviewed by Zhang [30]. In the Mesozoic, the lack of *in situ* sporomorphs is the main reason that the parent plants of dispersed sporomorphs can only be recovered at order or family level. The Eco-Plant model will be improved when more *in situ* sporomorphs are discovered. If the database is updated with data of new studies, the list of new publications will appear below the user interface (Fig. 1). Therefore, the users can verify how botanical affinities, EPH, and EPT for the sporomorph genera are derived.

The quality of sporomorph data in the uploaded dataset can also determine the quality of the result produced by the database. The genera included must be identified precisely by users using the appropriate genus names. All sporomorph names of taxa from different systems, fossil or extant, are governed by *the International Code of Nomenclature for algae, fungi, and plants* [94]. There are three parallel systems of sporomorph classification [95]: (1) natural, where reference to extant taxa is certain and the modern generic name could be used; (2) half-natural, where reference to an extant taxon is suspected but not proven; (3) artificial, where the relationship is not known at all, and a form-generic name based on morphological features is created. For this reason, different names may be used for the same sporomorph genus. In the Mesozoic, only half-natural and artificial systems are used. However, the differences between the two systems are not clear. Under the present code, “half-natural” names are just morphogenetic names and the same as “artificial” names, providing that they are validly published [95]. Because of the confusing nomenclature systems and the lack of a regulation about which name is the valid one, users should use the genus name with detailed descriptions and certain affinities when different names are available for the same sporomorph taxon. For example, *Cycadopites* Wodehouse 1933 ex Wilson et Webster 1946*,* has such a broad definition that it includes genera such as *Cycadaceaelagenella* Malyavkina 1953 (Cycadales) and *Ginkgocycadophytus* Samoilovich 1953 (Ginkgoales) [38,72,96]. In this case, *Cycadaceaelagenella* and *Ginkgocycadophytus* are advisable to be used rather than *Cycadopites*. For detailed identification of sporomorphs, sometimes SEM or transmission electron microscopy (TEM) are recommended since the specific characters of some pollen and spores can usually only be distinguished under SEM or TEM [4]. However, users can still use the in-advisable names, but they must keep such names as few as possible. In the results produced by the database, the botanical affinities, EPH, and EPT related with the in-advisable names will be marked as *Uncertain* and the sporomorphs have no contribution to the palaeoenvironmental analysis. The use of too many of such elements will make the result produced by the database insignificant. Advisable and in-advisable names for dispersed Mesozoic sporomorph genera of Bryophytes, Pteridophytes, and Gymnosperms are listed at http://www.sporopollen.com/sporefamilygenus.php?language=english.

The lack of *in situ* sporomorphs is also the main reason that the affinities of Paleozoic sporomorph taxa are currently not included in this database. Genera without certain affinities or which have not been included in the database will also be marked as *Uncertain* in the result for the botanical affinities, EPH, and EPT and reduce the quality of the result. Therefore, currently, the database is not suitable for records from the Paleozoic.

The database also provides a platform for possible cooperation. (1) Users are encouraged to contact the first author for including their published data in the datasets *Taxonomy* (Table 1) and *Ecogroup* (Table 2). (2) To date, almost all published sporomorph genera since the Early Triassic are included in the dataset *Taxonomy* (Table 1). However, if users find some genera that are still missing and the related references are available, they can contact the first author to update the database. (3) Suggestions for the improvement of the database by contacting the first author is always appreciated. In any case, the contributions of users to our database will always benefit the other users.

Supplementary material *and/or* Additional information: Appendix 1 PHP code for the user interface. Appendix 2 PHP and MySQL codes for different modes processing the uploaded dataset.

## Declaration of Competing Interest

The authors declare that they have no known competing financial interests or personal relationships that could have appeared to influence the work reported in this paper.

## Appendix Supplementary materials

Image, application 1

Image, application 2
