# Supplementary material for: Database-based Eco-Plant analysis for Mesozoic dispersed sporomorphs
Source: MethodsX. 2021 Apr 5;8:101329. doi: 10.1016/j.mex.2021.101329 (PMC8374400; doi:10.1016/j.mex.2021.101329)
Supplement: Supplementary file 1 [file mmc1.doc]

Appendix 1 PHP code for the user interface

$URL="TheProcessAddress";//This is the website address where the processing code is stored.

echo("<form id='drawplot' lang='en' target='_blank' name='drawplot' method='post' action='$URL'>");

echo("<input id='speciescombinationlist' name='speciescombinationlist' type='file' lang='en' value='open'></input>");

echo(" <select id='enenvironmenttype' name='enenvironmenttype'>");

echo("<option value='plant_family'>Vegetation Family</option>");

echo("<option value='plant_order'>Vegetation Order</option>");

echo("<option value='plant_phylum'>Vegetation Phylum</option>");

echo("<option value='humidity'>Eco-Plant EPH</option>");

echo("<option value='temperature'>Eco-Plant EPT</option>");

echo("<option value='plant_family_pdf'>Vegetation Family PDF</option>");

echo("<option value='plant_order_pdf'>Vegetation Order PDF</option>");

echo("<option value='plant_phylum_pdf'>Vegetation Phylum PDF</option>");

echo("<option value='humidity_pdf'>Eco-Plant EPH PDF</option>");

echo("<option value='temperature_pdf'>Eco-Plant EPT PDF</option>");

echo("<option value='drawcurve_pdf'>Draw Curve PDF</option>");

echo("</select>");

echo(" <input type='submit' value='Submit'></input>");

echo("</form>");
