# Supplementary material for: Database-based Eco-Plant analysis for Mesozoic dispersed sporomorphs
Source: MethodsX. 2021 Apr 5;8:101329. doi: 10.1016/j.mex.2021.101329 (PMC8374400; doi:10.1016/j.mex.2021.101329)
Supplement: Supplementary file 2 [file mmc2.doc]

Appendix 2 PHP and MySQL codes for different modes processing the uploaded dataset.

/*

This part includes the main functions of linking the user uploaded dataset with the datasets in this database and giving the result in dataset or diagram.

All the explanations are in orange. All the functional codes are in black.

As this database is designed for both Chinese and English users, some Chinese characters are shown in the codes.

*/

//Open MySQL database

$_DB=F_NetConnect();

if ($_DB==false){

return(false);

}

$outcropname=addslashes(trim($_POST["outcropname"]));

$enenvironmenttype=addslashes(trim($_POST["enenvironmenttype"]));

//Read the user-uploaded dataset

$fileobjectname="speciescombinationlist";

$ifwith0=false;

if ($enenvironmenttype=="drawcurve_pdf"){

$tablename=loadtcsvfile($fileobjectname,"","",true);

}else{

$tablename=loadtcsvfile($fileobjectname,"abundance,genus,sample","",true);

}

//If reading the uploaded dataset is false, exit

if ($tablename==false){

exit();

}

if($enenvironmenttype!="drawcurve_pdf"){

//Delete data that is not necessary

$query="delete from $tablename where genus is null or sample is null or genus='' or sample=''";

$result=mysql_query($query);

$query="delete from $tablename where abundance is null or abundance=0 or abundance=''";

$result=mysql_query($query);

$query="select genus from $tablename";

$result=mysql_query($query);

$num=mysql_num_rows($result);

if ($num==0){

echo("<br>错误! 您输入的数据表中没有有效的数据!!");

echo("<br>Error! There is no useful data in your uploaded dataset!!<br>");

}

//Transfer the uploaded numbers in to percentage.

//Count the number of samples

$query="select sample,sum(abundance) from $tablename group by sample order by sample";

$result=mysql_query($query);

$line=mysql_fetch_row($result);

$numlist="";

while ($line!=false){

if ($numlist==""){

$numlist="$line[1]";

$lastSample="$line[0]";

}else{

$numlist=$numlist.",$line[1]";

}

//next

$line=mysql_fetch_row($result);

}

$arrNum=explode(",",$numlist);

//Transfer percentage

$query2="select sample,genus,sum(abundance) from $tablename group by sample,genus order by sample,genus";

$result2=mysql_query($query2);

$num2=mysql_num_rows($result2);

$valuelist="";

if ($num2>0){

$line2=mysql_fetch_row($result2);

$n=0;

$kk=1;

while ($line2!=false){

if ($lastSample!=$line2[0]){

$lastSample=$line2[0];

$n=$n+1;

}

if ($arrNum[$n]=="0"){

$abundance=0;

}else{

$abundance=round((1000*$line2[2]) / $arrNum[$n])/10;

}

if ($valuelist==""){

$valuelist="('$line2[0]','$line2[1]','$abundance')";

}else{

$valuelist=$valuelist.",('$line2[0]','$line2[1]','$abundance')";

}

//next

$line2=mysql_fetch_row($result2);

$kk=$kk+1;

}

}

$query2="delete from $tablename";

$result2=mysql_query($query2);

$tablenameK=$tablename;

$tablename=randletter().$tablename;

$tablename=strtolower($tablename);

createtemptable($tablename,$querystring="",$idfield="PID",$noneidfieldlist="sample,genus,abundance");

$query2="insert into $tablename(sample,genus,abundance) values $valuelist";

$result2=mysql_query($query2);

//Calculate percentage

$sumtablename="sum".$tablename;

createtemptable($sumtablename,$querystring="select sample,genus,sum(abundance) as sumabundance from $tablename group by sample,genus order by sample,genus",$idfield="",$noneidfieldlist="");

//create genera list

$allgenustablename="allgenus".$tablename;

createtemptable($allgenustablename,$querystring="select distinct genus from $tablename order by genus",$idfield="",$noneidfieldlist="");

}

//Certify analysis mode

if (empty($enenvironmenttype)==true){

//exit if without useful data

echo("<script type='text/javascript'>window.location.href='../index.php';</script>" );

exit();

}

if (stripos($enenvironmenttype,"_pdf")>0){

$ifdrawPDF=true;

}else{

$ifdrawPDF=false;

}

if ($enenvironmenttype=="plant_family" or $enenvironmenttype=="plant_family_pdf"){

if (stripos($enenvironmenttype,"_pdf")==0){

echo("Vegetation Family 植被科<br>");

}

$datatablename=$sumtablename;

$proxytable="taxonomy";

$proxycolumn="family";

$withoutgenustablename="withoutg".$tablename;

$usefulgenustablename="useg".$tablename;

$resulttablename="result".$tablename;

reconstructsegs($datatablename,$proxytable,$proxycolumn,$withoutgenustablename,$usefulgenustablename,$resulttablename,$allgenustablename,$ifdrawPDF);

}else if ($enenvironmenttype=="plant_order" or $enenvironmenttype=="plant_order_pdf"){

if (stripos($enenvironmenttype,"_pdf")==0){

echo("Vegetation Order 植被目<br>");

}

$datatablename=$sumtablename;

$proxytable="taxonomy";

$proxycolumn="orders";

$withoutgenustablename="withoutg".$tablename;

$usefulgenustablename="useg".$tablename;

$resulttablename="result".$tablename;

reconstructsegs($datatablename,$proxytable,$proxycolumn,$withoutgenustablename,$usefulgenustablename,$resulttablename,$allgenustablename,$ifdrawPDF);

}else if ($enenvironmenttype=="plant_phylum" or $enenvironmenttype=="plant_phylum_pdf"){

if (stripos($enenvironmenttype,"_pdf")==0){

echo("Vegetation Phylum 植被门<br>");

}

$datatablename=$sumtablename;

$proxytable="taxonomy";

$proxycolumn="phylum";

$withoutgenustablename="withoutg".$tablename;

$usefulgenustablename="useg".$tablename;

$resulttablename="result".$tablename;

reconstructsegs($datatablename,$proxytable,$proxycolumn,$withoutgenustablename,$usefulgenustablename,$resulttablename,$allgenustablename,$ifdrawPDF);

}else if ($enenvironmenttype=="humidity" or $enenvironmenttype=="humidity_pdf"){

if (stripos($enenvironmenttype,"_pdf")==0){

echo("Eco-Plant EPH 湿度<br>");

}

$datatablename=$sumtablename;

$proxytable="humidity";

$proxycolumn="humidity";

$proxytable="taxonomy";

$proxycolumn="ephs";

$withoutgenustablename="withoutg".$tablename;

$usefulgenustablename="useg".$tablename;

$resulttablename="result".$tablename;

reconstructsegs($datatablename,$proxytable,$proxycolumn,$withoutgenustablename,$usefulgenustablename,$resulttablename,$allgenustablename,$ifdrawPDF);

}else if ($enenvironmenttype=="temperature" or $enenvironmenttype=="temperature_pdf"){

if (stripos($enenvironmenttype,"_pdf")==0){

echo("Eco-Plant EPT 温度<br>");

}

$datatablename=$sumtablename;

$proxytable="temperature";

$proxycolumn="temperature";

$proxytable="taxonomy";

$proxycolumn="ept";

$withoutgenustablename="withoutg".$tablename;

$usefulgenustablename="useg".$tablename;

$resulttablename="result".$tablename;

reconstructsegs($datatablename,$proxytable,$proxycolumn,$withoutgenustablename,$usefulgenustablename,$resulttablename,$allgenustablename,$ifdrawPDF);

}else if ($enenvironmenttype=="sealevel"){

if (stripos($enenvironmenttype,"_pdf")==0){

echo("Sea level 海平面<br>");

}

$datatablename=$sumtablename;

$proxytable="ecogroup";

$proxycolumn="ecogroup";

$withoutgenustablename="withoutg".$tablename;

$usefulgenustablename="useg".$tablename;

$resulttablename="result".$tablename;

reconstructsegs($datatablename,$proxytable,$proxycolumn,$withoutgenustablename,$usefulgenustablename,$resulttablename,$allgenustablename,$ifdrawPDF);

}else if ($enenvironmenttype=="plant_type"){

if (stripos($enenvironmenttype,"_pdf")==0){

echo("Vegetation Type 植被类型<br>");

}

$datatablename=$sumtablename;

$proxytable="genusplanttype";

$proxycolumn="planttype";

$withoutgenustablename="withoutg".$tablename;

$usefulgenustablename="useg".$tablename;

$resulttablename="result".$tablename;

reconstructsegs($datatablename,$proxytable,$proxycolumn,$withoutgenustablename,$usefulgenustablename,$resulttablename,$allgenustablename,$ifdrawPDF);

}else if ($enenvironmenttype=="drawcurve_pdf"){

$samplefieldname="sample";

if (table_fieldindex($tablename,$samplefieldname)<0){

$samplefieldname="Sample";

if (table_fieldindex($tablename,$samplefieldname)<0){

echo("<br>The Sample field is missing!<br>Sample列缺失!");

}

}

$DepthFieldName="depth";

if (table_fieldindex($tablename,$DepthFieldName)<0){

$DepthFieldName="Depth";

if (table_fieldindex($tablename,$DepthFieldName)<0){

$DepthFieldName="ID";

if (table_fieldindex($tablename,$DepthFieldName)<0){

$DepthFieldName="id";

}

}

}

DrawPollenCollumnPDF($tablename,$samplefieldname,$DepthFieldName);

}

function F_NetConnect($_说明_联接不成功返回false成功返回数据库联接标签=""){

//This function is to connect the MySQL database;

$_netConnect = mysql_connect(C_NI, C_NU, C_NP) ;

if ($_netConnect ==false) {

return($_netConnect);

}

// Connect database

$_DBConnect =mysql_select_db(C_ND) ;

if ($_DBConnect ==false) {

mysql_close($_netConnect);

return($_DBConnect);

}

//设置数据库字体这一步非常重要,否则会出现乱码

mysql_query("set names utf8");

//返回值

return($_netConnect);

}

function loadtcsvfile($csvobjectname,$fieldmustinclude="", $newtablename="",$iftemptable=false){

//说明 $csvobjectname 被上传文件在表单中的名称

//说明 $fieldmustinclude 被上传文中必须包含的列加逗号分隔,如果为空说明所有类型都可以, 如果被上传文件不满足要求则返回false函数结束

//说明 $newtablename 被上传文件被上传后的表名称,如果为空则为: 随机数组+IP+随机字母+时间 构成

//说明 $iftemptable 文件上传后是否形成临时文件, 是值为true,不是值为false,默认值为false.

//说明 函数调用不成功返回false,调用成功返回新生成表名.

//打开数据库

$_DB=F_NetConnect();

if ($_DB==false){

if (Language=="简体中文"){

echo("信息:数据库无法打开!!!");

}else{

echo("信息:database can not be open!!!");

}

exit();

}

set_time_limit(0);

// 导入CSV文件必须有 Genus列

//将生成临时表,如果不成功返回false 成功返回生成表名

//fieldmustinclude 为一定要包含的列列表逗号分隔

//newtablename 空为随机名称

//iftemptable=true 为新建临时表

//有错退出

if($_FILES["error"] != 0){

return(false);

}

if(empty($_FILES[$csvobjectname]["tmp_name"])==true){

return(false);

}

//确保文件格式正确

//提取文件后缀

$pos=strrpos($_FILES[$csvobjectname]["name"],".");

if ($pos==0 or $pos==false){

return(false);

}

$suffix=substr($_FILES[$csvobjectname]["name"],$pos);

$suffix=trim($suffix);

//文件后缀不是csv退出

if ($suffix!=".csv" ){//只允许上传csv格式文件

return(false);

}

//提取首列

$file=fopen($_FILES[$csvobjectname]["tmp_name"],"r");//打开文件

fseek($file,0);//移到文件首

$line=fgetcsv($file);//获取第一行各列数组

$line[0]=iconv('gb2312','utf-8',$line[0]);//将所行数据转换为通用代码

$mm=count($line);//总列数

$ifbreackbycomma=true;

if ($mm==1 and stripos($line[0],";")>0){//某些文件分号分行必须重新分行

$line=explode(";",$line[0]);

$mm=count($line);

$ifbreackbycomma=false;

}

//获致所有列列表

$nn=0;

$allfieldlist=",";

while($nn<$mm){

if ($nn!=0 and $ifbreackbycomma=true){

$line[$nn]=iconv('gb2312','utf-8',$line[$nn]);

}

$line[$nn]=trim($line[$nn]);

$line[$nn]=strtolower($line[$nn]);

$allfieldlist=$allfieldlist.$line[$nn].",";

//下一步

$nn=$nn+1;

}

$allfieldlist=",".$allfieldlist.",";

//确定必有列存在

$mustfieldlist=$fieldmustinclude;

$mustfieldlist=str_replace(" ","",$mustfieldlist);

if (empty($mustfieldlist)==false){

$field=explode(",",$mustfieldlist);

$n=0;

$m=count($field);

while ($n<$m){

$k=stripos($allfieldlist,",".$field[$n].",");

if ($k==false or $k<1){

echo("<br>错误!!数据输入表缺少 $field[$n] 列 !!<br> Error!!Uploaded dataset must includes conlumn $field[$n] !!");

return(false);

}

$n=$n+1;

}

}

//恢复所有列列表

$allfieldlist=str_replace(",,","",$allfieldlist);

//新建表

//确定文件名

$tablename=trim($newtablename);

if (empty($tablename)==true){

$tablename=randletter().$_SERVER['REMOTE_ADDR'].randletter().date("YmdHis");

$tablename=str_replace(".","",$tablename);

}

//新建表

if ($iftemptable==true){

createtemptable($tablename,$querystring="",$idfield="ID",$noneidfieldlist=$allfieldlist);

}else{

createtable($tablename,$querystring="",$idfield="ID",$noneidfieldlist=$allfieldlist);

}

//填充数据

$line=fgetcsv($file);

while ($line!=false){

//数据处理

$line[0]=iconv('gb2312','utf-8',$line[0]);

$mm=count($line);//总列数

$ifbreackbycomma=true;

if ($mm==1 and stripos($line[0],";")>0){

$line=explode(";",$line[0]);

$mm=count($line);

$ifbreackbycomma=false;

}

//提取数据

$nn=0;

$value="";

while ($nn<$mm){

if ($nn!=0 and $ifbreackbycomma=true){

$line[$nn]=iconv('gb2312','utf-8',$line[$nn]);

}

$line[$nn]=trim($line[$nn]);

if ($value==""){

$value="'".$line[$nn]."'";

}else{

$value=$value.",'".$line[$nn]."'";

}

//下一步

$nn=$nn+1;

}

//导入数据表

$query="insert into ".$tablename."(".$allfieldlist.") values(".$value.")";

mysql_query($query);

//下一步

$line=fgetcsv($file);

}

//返回临时表名

return($tablename);

}

function randletter(){//返回随机大写字母

$num=mt_rand(65,90);

$str=chr($num);

return($str);

}

function createtemptable($tablename,$querystring="",$idfield="",$noneidfieldlist=""){

//$query为空时id列,普通列都必须存在 $query不为空时时id列,普通列无效

//新建临时表

//打开数据库

$_DB=F_NetConnect();

if ($_DB==false){

return(false);

}

//数据处理

//表名

$str=trim($tablename);

if (empty($str)==true){

return(false);

}

$query= "create temporary table ".$str."(";

//查询字符串

$str=trim($querystring);

if (empty($str)==false){

$query=$query.$str.")";

$result = mysql_query($query);

//返回是否调用成功

if ($result==true){

$r=true;

return($r);

}else{

$r=false;

return($r);

}

}

//ID列名

$str=trim($idfield);

if (empty($str)==true){

return(false);

}

$query= $query.$str." bigint AUTO_INCREMENT";

//普通列

$str=trim($noneidfieldlist);

if (empty($str)==true){

return(false);

}

$arr=explode(",",$str);

$m=count($arr);

$n=0;

while ($n<$m){

$query=$query.",".$arr[$n]." text CHARACTER SET utf8 COLLATE utf8_unicode_ci";

$n=$n+1;

}

//key ID列名

$str=trim($idfield);

if (empty($str)==true){

return(false);

}

$query= $query.",PRIMARY KEY (".$str."))";

//建立表格

$result = mysql_query($query);

//返回是否调用成功

if ($result==true){

$r=true;

return($r);

}else{

$r=false;

return($r);

}

}

function reconstructsegs ($datatablename, $proxytable, $proxycolumn, $withoutgenustablename, $usefulgenustablename, $resulttablename, $allgenustablename, $ifdrawPDF=false){

//说明 $datatablename 被分析数据表必需同时包含 genus, abundance, sample 三列

//说明 $proxytable 存放媒介表比如 humidity

//说明 $proxycolumn 存和媒介列比如 humit

//说明 $withoutgenustablename 生成无内容属表名

//说明 $usefulgenustablename 生成有内容属表名

//说明 $resulttablename 生成结果表名

//生成有用属表

$querystring="select distinct ".$proxytable.".".$proxycolumn.",".$proxytable.".genus from ".$proxytable." inner join ".$allgenustablename." on ".$proxytable.".genus=".$allgenustablename.".genus order by ".$proxycolumn;

createtemptable($usefulgenustablename,$querystring,$idfield="",$noneidfieldlist="");

//生成无用属表

$querystring="select distinct ".$allgenustablename.".genus from ".$allgenustablename." left join ".$usefulgenustablename." on ".$usefulgenustablename.".genus=".$allgenustablename.".genus where ".$usefulgenustablename.".genus is null";

createtemptable($withoutgenustablename,$querystring,$idfield="",$noneidfieldlist="");

//生成未交叉结果表

$resulttablename0="zz".$resulttablename;

$querystring="select ".$datatablename.".sample,".$usefulgenustablename.".".$proxycolumn.",sum(".$datatablename.".sumabundance) as abundance from ".$datatablename." left join ".$usefulgenustablename." on ".$datatablename.".genus=".$usefulgenustablename.".genus group by ".$datatablename.".sample,".$usefulgenustablename.".".$proxycolumn."";

createtemptable($resulttablename0,$querystring,$idfield="",$noneidfieldlist="");

$querystring="update ".$resulttablename0." set ".$proxycolumn."='Uncertain' where ".$proxycolumn." is null or ".$proxycolumn."=''";

$result=mysql_query($querystring);

//生成交叉结果表

transformtable($resulttablename0,$groupfieldlist="sample",$pivotfield=$proxycolumn,$valuefield='abundance',$resulttablename,$iftemp=true,$wherewithoutwhere="",$calculate="sum",$nulldefault="0");

//显示结果

if ($ifdrawPDF==true){

DrawPollenCollumnPDF($resulttablename,"sample","id");

}else{

echo("<br>Result 分析结果<br>");

showtablealldata($resulttablename);

echo("<br>This Result is based on the following genera collected in this database.<br>");

echo("本分析结果根据本数据库已收集到的如下指示化石得出.<br>");

showtablealldata($usefulgenustablename);

$query="select genus from ".$withoutgenustablename." ";

$result=mysql_query($query);

$num=mysql_num_rows($result);

if ($num==1){

echo("<br>As the environmental significance of following genus is not clear, its abundance is under the column uncertain.<br>");

echo("以下属的环境意义尚不表楚,其丰度值位于结果的uncertain列<br>");

showtablealldata($withoutgenustablename);

}else if ($num>1){

echo("<br>As the environmental significances of following genera are not clear, their total abundance is under the column uncertain.<br>");

echo("以下属的环境意义尚不表楚,其丰度值位于结果的uncertain列<br>");

showtablealldata($withoutgenustablename);

}

}

}

function DrawPollenCollumnPDF($TableWithAbundance,$SampleFieldName,$DepthFieldName,$AverageColumnWidthMm=10,$NotShowFieldList=""){

$HeightEachRowMm=4;

$GapEachColumnMm=1;

$MarginMm=20;

$pix_mm=360/127;;

//没有深度列则id列为深度

$SampleFieldName=str_replace(",","",$SampleFieldName);

$NotShowFieldList=str_replace(",","",$NotShowFieldList);

//提取内容

$querystring="select * from $TableWithAbundance";

$result=mysql_query($querystring);

$num=mysql_num_rows($result);

$FieldNum=mysql_num_fields($result);

if ($num==0){

return(-1);

}

$DrawHeightMm=$num*$HeightEachRowMm;

//提取所有列 数值列

$n=0;

$AllFiledList="";

$ValueFieldlist="";

$MaxValueFieldlist="";

$DepthFieldIndex=-1;

while ($n<$FieldNum){

$FieldName=mysql_field_name($result,$n);

if ($FieldName==$DepthFieldName){

$DepthFieldIndex=$n;

}

if ($AllFiledList==""){

$AllFiledList=$FieldName;

}else{

$AllFiledList=$AllFiledList.",".$FieldName;

}

if (stripos(",,".$DepthFieldName.",".$SampleFieldName.",".$NotShowFieldList,",".$FieldName.",")==0){

if ($ValueFieldlist==""){

$ValueFieldlist=$FieldName;

$MaxValueFieldlist="max(CONVERT(".$FieldName.", DECIMAL(10,2)))";

}else{

$ValueFieldlist=$ValueFieldlist.",".$FieldName;

$MaxValueFieldlist=$MaxValueFieldlist.",max(CONVERT(".$FieldName.", DECIMAL(10,2)))";

}

}

$n=$n+1;

}

if ($DepthFieldIndex==-1){

return(-1);

}

//计算数值列总宽度

$querystring="select $MaxValueFieldlist from $TableWithAbundance";

$MaxResult=mysql_query($querystring);

$numValueField=mysql_num_fields($MaxResult);

$AllValueSum=0;

$MaxValue=0;

$MinValue=0;

$AllValueList="";

$MaxLine=mysql_fetch_row($MaxResult);

$n=0;

while ($n<$numValueField){

$AllValueSum=$AllValueSum+$MaxLine[$n];

if ($AllValueList==""){

$AllValueList=$MaxLine[$n];

}else{

$AllValueList=$AllValueList.",".$MaxLine[$n];

}

if ($MaxLine[$n]>$MaxValue){

$MaxValue=$MaxLine[$n];

}

if ($MinValue==0){

$MinValue=$MaxLine[$n];

}else if ($MinValue>$MaxLine[$n]){

$MinValue=$MaxLine[$n];

}

//next

$n=$n+1;

}

$MeanValue=$AllValueSum/$numValueField;

//计算绘图列总宽度

$n=0;

$DrawAllValueSum=0;

while ($n<$numValueField){

if ($MaxLine[$n]<$MeanValue/2){

$CurrDrawValue=$MeanValue/2;

}else{

$CurrDrawValue=$MaxLine[$n]+$MeanValue/10;

}

$DrawAllValueSum=$DrawAllValueSum+$CurrDrawValue;

//next

$n=$n+1;

}

//计算标本列总宽度

$index=query_fieldindex($result,$SampleFieldName);

mysql_data_seek($result, 0);

$line=mysql_fetch_row($result);

$MaxSampleNameMm=0;

$fonttype="./fonts/arialuni.ttf";

if (file_exists($fonttype)==false){

if (file_exists("../fonts/arialuni.ttf")==true){

$fonttype = "../fonts/arialuni.ttf";

}else{

$fonttype = "./fonts/arialuni.ttf";

}

}

$fontsize=8;

while ($line!=false){

$str=$line[$index];

$alltextpixsize=imagettfbbox($fontsize,0,$fonttype,$str);

$strPixSize=$alltextpixsize[2]-$alltextpixsize[0];

$strPixSize=$strPixSize/$pix_mm;

if ($MaxSampleNameMm<$strPixSize){

$MaxSampleNameMm=$strPixSize;

}

//next

$line=mysql_fetch_row($result);

}

$DrawWeidthMm=$MaxSampleNameMm+$DrawAllValueSum*$AverageColumnWidthMm/$MeanValue+$GapEachColumnMm*($numValueField+1);

$PdfWeidthMm=$DrawWeidthMm+3*$MarginMm;

$PdfHeightMm=$DrawHeightMm+3*$MarginMm;

//新建PDF

$pdf=new PDF_Extend();

if ($PdfHeightMm>$PdfWeidthMm){

$pdf->__construct("P","mm",array($PdfWeidthMm,$PdfHeightMm));

}else{

$pdf->__construct("L","mm",array($PdfWeidthMm,$PdfHeightMm));

}

$pdf->AddPage();

$pdf->SetFont('Arial','',10);

/* 参数

$TextString=$ValueFieldlist;

//$pdf->Write(10,$MaxValueFieldlist);

$pdf->Write(10,$AllValueList);

$pdf->Write(10,chr(10)."$numValueField All Values: $AllValueSum");

$pdf->Write(10,chr(10)."DrawAllValueSum: $DrawAllValueSum");

$pdf->Write(10,chr(10)."MaxSampleNameMm: $MaxSampleNameMm");

$pdf->Write(10,chr(10)."Min Value: $MinValue");

$pdf->Write(10,chr(10)."Max Value: $MaxValue");

$pdf->Write(10,chr(10)."Mean Value: $MeanValue");

$pdf->RotateText($TextString,$WordX=10,$WordY=140,$WordSize=10,$WordAngle=0,$WordColorR=0,$WordColorG=0,$WordColerB=0,$WordFamily="Arial",$WordStyle="");

//输出PDF

*/

//标出标本

mysql_data_seek($result, 0);

$line=mysql_fetch_row($result);

$n=1;

while ($line!=false){

$str=$line[$index];

$alltextpixsize=imagettfbbox($fontsize,0,$fonttype,$str);

$strPixSizeY=$alltextpixsize[1]-$alltextpixsize[7];

$strSizeYMm=$strPixSizeY/$pix_mm;

$CurrY=2*$MarginMm+$DrawHeightMm-$n*$HeightEachRowMm+$HeightEachRowMm/2+$strSizeYMm/2;

$pdf->RotateText($str,$WordX=$MarginMm,$WordY=$CurrY,$fontsize,$WordAngle=0,$WordColorR=0,$WordColorG=0,$WordColerB=0,$WordFamily="Arial",$WordStyle="");

//next

$line=mysql_fetch_row($result);

$n=$n+1;

}

//标出标本线

$n=1;

$SampleScaleStyle = array('width' => 0.2, 'cap' => 'butt', 'join' => 'miter', 'dash' => '0', 'phase' => 10,'color' => array(232, 232, 232));

$SampleXL=$MarginMm+$MaxSampleNameMm;

$SampleXR=$MarginMm+$DrawWeidthMm;

while ($n<=$num){

$SampleY=2*$MarginMm+$DrawHeightMm-$n*$HeightEachRowMm+$HeightEachRowMm/2;

$pdf->Line($SampleXL, $SampleY, $SampleXR, $SampleY, $SampleScaleStyle);

//next

$n=$n+1;

}

//标出方格

$n=0;

$DrawAllValueSum=0;

$LastXmm=$MarginMm+$MaxSampleNameMm;

$TopYMm=2*$MarginMm;

$BottomYMm=$TopYMm+$DrawHeightMm;

$ValueFieldNameArr=explode(",",$ValueFieldlist);

$BoxLineStyle = array('width' => 0.2, 'cap' => 'butt', 'join' => 'miter', 'dash' => '0', 'phase' => 10,'color' => array(0, 0, 0));

while ($n<$numValueField){

//计算当前列宽

if ($MaxLine[$n]<$MeanValue/2){

$CurrDrawValue=$MeanValue/2;

}else{

$CurrDrawValue=$MaxLine[$n]+$MeanValue/10;

}

$CurrDrawWidthMm=$AverageColumnWidthMm*$CurrDrawValue/$MeanValue;

$CurrXMm=$GapEachColumnMm+$LastXmm+$CurrDrawWidthMm;

//标出列名

$pdf->RotateText($ValueFieldNameArr[$n],$WordX=2*$GapEachColumnMm+$LastXmm,$WordY=$TopYMm-$GapEachColumnMm,$WordSize=10,$WordAngle=30,$WordColorR=0,$WordColorG=0,$WordColerB=0,$WordFamily="Arial",$WordStyle="");

//画左线

$pdf->Line($GapEachColumnMm+$LastXmm, $TopYMm, $GapEachColumnMm+$LastXmm, $BottomYMm, $BoxLineStyle);

//画右线

$pdf->Line($CurrXMm, $TopYMm, $CurrXMm, $BottomYMm, $BoxLineStyle);

//画上线

$pdf->Line($GapEachColumnMm+$LastXmm, $TopYMm, $CurrXMm, $TopYMm, $BoxLineStyle);

//画下线

$pdf->Line($GapEachColumnMm+$LastXmm, $BottomYMm, $CurrXMm, $BottomYMm, $BoxLineStyle);

//设置刻度

$imagepixrange=$CurrDrawWidthMm*$pix_mm;

$reallength=$CurrDrawValue;

$realsmallscale=calculatesmallscale($imagepixrange,$reallength);

$realbigscale=5*$realsmallscale;

$donotshowrealbigscale="-10";

$axiszonerealXleft=0;

$axiszonerealXright=$CurrDrawValue;

$axiszonerealYright=0.5;

$axiszonerealYleft=0.5;

$axisrealY=0.5;

$drawzonelefttoppixX=($GapEachColumnMm+$LastXmm)*$pix_mm;

$drawzonelefttoppixY=$TopYMm*$pix_mm;

$drawzonerightbottompixX=$CurrXMm*$pix_mm;

$drawzonerightbottompixY=$BottomYMm*$pix_mm;

$drawzonelefttoprealX=0;

$drawzonerightbottomrealX=$CurrDrawValue;

$drawzonelefttoprealY=$num+0.5;

$drawzonerightbottomrealY=0.5;

PdfDrawScaleX($pdf,$realsmallscale,$realbigscale,$donotshowrealbigscale,$axisrealY,$axiszonerealXleft,$axiszonerealYleft,$axiszonerealXright,$axiszonerealYright,$drawzonelefttoppixX,$drawzonelefttoppixY,$drawzonerightbottompixX,$drawzonerightbottompixY,$drawzonelefttoprealX,$drawzonelefttoprealY,$drawzonerightbottomrealX,$drawzonerightbottomrealY,$ifisupscale=true,$ifdrawbigscale=true,$startcaption="",$endcaption="");

//画多边形

//提取多边形坐标

mysql_data_seek($result, 0);

$line=mysql_fetch_row($result);

$nn=1;

$ValueFieldInext=query_fieldindex($result,$ValueFieldNameArr[$n]);

$DXMm=$GapEachColumnMm+$LastXmm;

$SampleY=2*$MarginMm+$DrawHeightMm-$nn*$HeightEachRowMm+$HeightEachRowMm/2;

$ArrStr=$DXMm.",".$SampleY;

while ($line!=false){

$CurrAbundance=$line[$ValueFieldInext];

$CurrPolyLineWidthMm=$AverageColumnWidthMm*$CurrAbundance/$MeanValue;

$SampleY=2*$MarginMm+$DrawHeightMm-$nn*$HeightEachRowMm+$HeightEachRowMm/2;

$DXMm=$GapEachColumnMm+$LastXmm+$CurrPolyLineWidthMm;

$ArrStr=$ArrStr.",".$DXMm.",".$SampleY;

//next

$line=mysql_fetch_row($result);

$nn=$nn+1;

}

$DXMm=$GapEachColumnMm+$LastXmm;

$ArrStr=$ArrStr.",".$DXMm.",".$SampleY;

$PolyArr=explode(",",$ArrStr);

//绘制多边形

$pdf->Polygon($PolyArr, 'F',array('all' => $BoxLineStyle),array(0,0,0));

//next

$n=$n+1;

$LastXmm=$CurrXMm;

}

$pdf->Output();

return(-1);

}
